# Changes in miRNA expression in patients with peripheral arterial vascular disease during moderate- and vigorous-intensity physical activity

**DOI:** 10.1007/s00421-022-05091-2

**Published:** 2022-11-23

**Authors:** Johanna Sieland, Daniel Niederer, Tobias Engeroff, Lutz Vogt, Christian Troidl, Thomas Schmitz-Rixen, Winfried Banzer, Kerstin Troidl

**Affiliations:** 1 Department of Sports Medicine, Institute of Sports Sciences, Goethe University, Ginnheimer Landstraße 39, 60487 Frankfurt, Germany; 2 Institute for Occupational Medicine, Social Medicine and Environmental Medicine, Division Health and Performance, Goethe University, Theodor-Stern-Kai 7, 60590 Frankfurt, Germany; 3 Department of Experimental Cardiology, Medical Faculty, Justus-Liebig-University, 35392 Giessen, Germany; 4 Department of Cardiology, Kerckhoff Heart and Thorax Center, 61231 Bad Nauheim, Germany; 5 German Center for Cardiovascular Research (DZHK), Partner Site RheinMain, Frankfurt Am Main, Germany; 6 Department of Vascular and Endovascular Surgery, University Hospital Frankfurt, Theodor-Stern-Kai 7, 60590 Frankfurt, Germany; 7 Department of Life Sciences and Engineering, TH Bingen, Berlinstrasse 109, 55411 Bingen Am Rhein, Germany; 8 Division of Preventive and Sports Medicine, Institute for Occupational Medicine, Social Medicine and Environmental Medicine, Goethe University, Theodor-Stern-Kai 7, 60590 Frankfurt, Germany

**Keywords:** Circulating miRNA, miRNA-142-5p, miRNA-424-5p, Walking training, Arteriogenesis, Lower extremity arterial disease

## Abstract

**Background:**

Walking is the preferred therapy for peripheral arterial disease in early stage. An effect of walking exercise is the increase of blood flow and fluid shear stress, leading, triggered by arteriogenesis, to the formation of collateral blood vessels. Circulating micro-RNA may act as an important information transmitter in this process. We investigated the acute effects of a single bout of 1) aerobic walking with moderate intensity; and 2) anaerobic walking with vigorous intensity on miRNA parameters related to vascular collateral formation.

**Methods:**

Ten (10) patients with peripheral arterial disease with claudication (age 72 ± 7 years) participated in this two-armed, randomized-balanced cross-over study. The intervention arms were single bouts of supervised walking training at (1) vigorous intensity on a treadmill up to volitional exhaustion and (2) moderate intensity with individual selected speed for a duration of 20 min. One week of washout was maintained between the arms. During each intervention, heart rate was continuously monitored. Acute effects on circulating miRNAs and lactate concentration were determined using pre- and post-intervention measurement comparisons.

**Results:**

Vigorous-intensity walking resulted in a higher heart rate (125 ± 21 bpm) than the moderate-intensity intervention (88 ± 9 bpm) (*p* < 0.05). Lactate concentration was increased after vigorous-intensity walking (*p* = 0.005; 3.3 ± 1.2 mmol/l), but not after moderate exercising (*p* > 0.05; 1.7 ± 0.6 mmol/l). The circulating levels of miR-142-5p and miR-424-5p were up-regulated after moderate-intensity (*p* < 0.05), but not after vigorous-intensity training (*p* > 0.05).

**Conclusion:**

Moderate-intensity walking seems to be more feasible than vigorous exercises to induce changes of blood flow and endurance training-related miRNAs in patients with peripheral arterial disease. Our data thus indicates that effect mechanisms might follow an optimal rather than a maximal dose response relation. Steady state walking without the necessity to reach exhaustion seems to be better suited as stimulus.

**Supplementary Information:**

The online version contains supplementary material available at 10.1007/s00421-022-05091-2.

## Introduction

Cardiovascular diseases are some the most common causes of death (Heil et al. 2006; Bjarnason-Wehrens et al. 2007). One of these pathologies is peripheral artery disease, which is a major cause of cardiovascular morbidity and mortality (Murray et al. 2012). Despite the widespread availability and use of effective risk-reducing interventions, the burden of this disease is increasing worldwide (Emdin et al. 2015). The prevalence of peripheral arterial disease for the overall population is estimated to be around 202 million worldwide (Lawall et al. 2016) leading to a proportion of > 15% diseased in the age range over 70 years (Espinola-Klein et al. 2019).

Peripheral arterial disease, inter alia, impairs lower limb performance (Dopheide et al. 2017). In particular walking performance of patients, operationalised by walking distance, can be reduced by more than 50% (Barker et al. 2004). In addition, peak oxygen uptake in persons with intermittent claudication is approximately 50% less than in the normal population (Barker et al. 2004). These restrictions lead to an impact on one’s performance during activities of daily living and to significant impairments in health-related quality of life (Guidon and McGee 2010; McDermott et al. 2013). In peripheral arterial disease with claudication, the reduced blood flow in the arteries supplying the lower extremities leads to a reduced supply of oxygen to the working muscles while walking (Schaardenburgh et al. 2017). Typically, patients complain of claudication pain in the calves, which releases after a short break (Fokkenrood et al. 2013).

Supervised exercise therapies aim to improve quality of life as well as lower limb symptoms and performance. Therapy options in non-clinical settings include home-based walking and resistance exercises (Guidon and McGee 2010; McDermott et al. 2013; Morris et al. 2014). Walking training is the preferred conservative form of therapy for patients with peripheral arterial disease with claudication (Murphy et al. 2015). Pain-free and maximum walking distance can dramatically be increased through supervised regularly performed walking training (Barker et al. 2004; Erbel and Katus 2019).

The exact cellular and molecular mechanisms leading to these performance improvements have not yet been unravelled (Düppers et al. 2019). Immediately after exercise, a more effective distribution of blood (Watson et al. 2018) and an increase in the diameter of collateral arteries, among other things, could be measured (Espinola-Klein et al. 2019). In addition to factors influencing blood flow, symptoms might also be diminished by an economisation effect concerning metabolism and intermuscular coordination (Remijnse-Tamerius et al. 1999). This may results in lower oxygen demands during matched walking performance, vice versa leading to an improvement in walking ability (Bresler et al. 2019).

Long-term effects on increased blood flow may be mostly attributed to arteriogenesis, in particular the formation of collateral blood vessels to bypass a stenosis or occlusion (Tan et al. 2000). Based on increased circulation, one of the main influencing factors for lower limb arteriogenesis is physical activity (Vogel et al. 2020). Blood flow, diverted into the collateral arteries by an existing stenosis, is the trigger to initiate arteriogenesis. The increased blood flow in the collaterals and the accompanying altered shear stress are the initial stimulus for long-term vessel remodelling and diameter growth (Vogel et al. 2020). The expansion of collateral arteries creates a natural bypass that increases blood flow to hypoxic tissue (Green et al. 2017). Within the pathway from increased fluid shear stress to collateral formation, microRNAs (miRNAs) may act as important information transmitters filling the gap between acute reactions of nitric oxide and ion channels and long-term morphologic adaptations. Since cardiovascular adaptations are discussed to improve with increasing levels of intensity (MacInnis and Gibala 2017), vigorous-intensity supervised exercise in patients with peripheral arterial disease might lead to greater vascular adaptations.

The sketched mechanistic is mainly seen in healthy people. In these individuals’, cardiovascular adaptions improve with higher exercise intensity. However, it is currently unclear whether these adaptions occur in patients with PAD. Although initially seen as by-product of anaerobic metabolism or fuel for specific muscle cells, lactate is currently discussed as transmitter or signaling molecule for processes including angiogenesis (Porporato et al. 2012) and compensatory cardiorespiratory reflexes (Torres-Torrelo et al. 2021). Lactate concentrations during exercise in patients with peripheral arterial disease differ in comparison to control groups (Parr et al. 2008). Furthermore, a higher lactate cumulation was associated with the development of leg pain, which is often the limitation of walking performance in patients with peripheral arterial disease (Parr et al. 2008). Therefore, the lactate concentration could be an important variability in exercise performance of patients with peripheral arterial disease and even might act as stimulus for underlying mechanisms of arteriogenesis.

The background of, inter alia, the relevance of investigating is the role of miRNA in the therapy of peripheral arterial disease. Based on recent evidence, miRNAs could be developed as potential circulating biomarkers for the diagnosis and prognosis of PAD (Bresler et al. 2019; Sayed et al. 2010; Håkansson et al. 2018). Potential regulations of miRNAs during exercises (at different intensities) could deliver a first hint on the potential association of acute vascular and endothelial response, miRNA expressions and beneficial exercise effects on arteriogenesis. In healthy persons, an up-regulation of the miR-142-5p, miR-197-3p, miR-342-3p and miR-424-5p is already documented for moderate-intensity training, vigorous-intensity training or artificially blood flow restricted situations, as identified in a screen targeting 179 human plasma-relevant miRNAs (Sieland et al. 2021). Additionally, a correlation between lactate concentration and miR-99a-5p, miR-199a-3p and miR125b-5p is documented (Sieland et al. 2021). These regulations may act as a model situation for peripheral arterial disease mechanisms (Vogel et al. 2020). A translation to persons suffering from peripheral arterial disease has not yet been done.

The aim of the present study was to investigate the influence of walking exercise itself and different exercise intensities on cardiometabolic outcomes and the expression of miRNAs which are related to short term vascular response and arteriogenesis in patients with peripheral arterial disease. We hypothesise that vigorous-intensity training leads to a higher metabolic and to a different profile of circulating miRNAs compared to moderate-intensity training.

## Materials and methods

### Ethical standard and study design

The study adopts a randomized-balanced crossover design. Ethical approval was obtained from the local independent institutional review board (protocol-number 2019–04, 05.03.2019, Ethics Committee Department 5 Psychology and Sports Sciences Goethe-University Frankfurt). The trial was conducted in accordance with the ethical standards set down by the declaration of Helsinki (World medical Association Declaration of Helsinki–Ethical Principles for Medical Research Involving Human Subjects) with its recent modification of 2013 (Fortaleza). All participants gave written informed consent prior to study enrolment.

### Sample

Adults (> 17 years of age) of any sex/gender were included. Participants had to have diagnostically confirmed peripheral arterial disease of any grade and claudication. Exclusion criteria included (1) critical limb ischemia, (2) acute infection or severe illnesses that have an impact on quality of life or physical performance, (3) use of perception-altering substances, (4) muscle soreness or severe pain in the lower extremities, (5) existing pregnancy or breastfeeding period.

### Experimental design

The cross-sectional study consisted two visits (on two different days with a washout period of at least seven days), in a randomized, balanced sequence. Visit A was the vigorous-intensity intervention and included a treadmill walking intervention with gradual increase in the angle of ascent until volitional exhaustion. Visit B, the moderate-intensity intervention involved guided walking training at individual walking speed. During each training intervention, loading-associated outcomes were monitored. The acute effects were determined using pre- and post-intervention measurements.

### Intervention

In the vigorous-intensity intervention, participants executed a step incremental walking exercise until volitional exhaustion on a treadmill. The intervention was based on to the incremental Gardner-Skinner protocol (Gardner et al. 2007). Initial treadmill speed was 0.89 m/s at an incline of 0%. After each 2 min-stage, the incline was increased by 2%. Heart rate was recorded at the end of each stage. Usually, the load is performed to the point of full exertion (Heck 2001). In case of patients with peripheral arterial disease, volitional exhaustion is reached based on ischaemia in the lower limb and resulting pain which leads to termination before exertion of other physiological factors. The assessment was, therefore, terminated as soon as a severe impairment of walking performance due to pain in the legs or other termination criteria such as shortness of breath occurred. The exercise, therefore, comprised of a single bout with standardized intensity but individualized duration until maximal effort. The intervention aimed at inducing a single but maximal metabolic stimulus.

The moderate-intensity intervention was conducted as an interval-training with standardized duration and individualized intensity. This exercise therefore aimed at mimicking real-life situations during habitual behaviours (such as health enhancing physical activity or activities of daily living). Participants were instructed to walk a standardized track multiple times within 20 min. The subjects selected their maximum gait speed, which they were able to maintain constantly over 20 min. In case of pain in the legs, the participants were allowed to take a short break at any time. The claudication pain usually subsides quickly at rest, so a short break is characteristically during walking exercises. On each test day, a standardized control condition (“do-nothing” phase) was performed for 20 min prior to the beginning of the testing condition.

### Outcomes

#### Blood lactate concentration

Before and directly after each intervention, capillary blood was taken by pricking the earlobe with a safety lancet. The sample was applied directly (maximal 30 s after intervention) to a test strip to determine lactate concentration (mmol/l) by means of a portable, hand-held unit (Lactate Scout, SensLab GmbH, Leipzig, Germany). The unit proves to be a reliable lactate measurement system with an inter-rater reliability with a correlation coefficient of *r* = 0.95 for inter-rater reliability and *r* = 0.91 for intra-rater reliability (Tanner et al. 2010). The Lactate Scout is reported to have 99% accuracy and 98% precision (Kaynar et al. 2015).

#### Heart rate

During the intervention, a chest belt (Polar H7) and heart rate receiver (Polar M 430) continuously measured heart rate (beats/min). The maximum heart rate was selected for further analyses.

#### miRNA profiling

##### Blood sampling and plasma preparation for miRNA quantification

To minimize pre-analytical variables that might influence the miRNA expression profile, collection of blood and the preparation of plasma was conducted with a minimal risk of blood cell contamination and haemolysis. Before and after each intervention, fingertip capillary blood samples (≥ 200 μL) were collected in microvettes (system for capillary blood collection) containing Ethylenediaminetetraacetic acid (EDTA). Blood samples were centrifuged for 10 min at 3000 rpm and 4 °C. After the first centrifugation step, the upper plasma phase was transferred to a new tube without disturbing the intermediate buffy coat layer. The plasma samples were centrifuged a second time for 10 min at 15,000 rpm and 4 °C. The cleared supernatant was carefully transferred to a new tube and frozen at − 80 °C.

##### miRNA isolation

Sample amounts were standardized by volume: The same volume of plasma was used for each RNA isolation, and the same volume of purified RNA was used for all further analyses. The miRNAs were isolated from 50 µL of plasma using a column-based protocol (miRNeasy Serum/Plasma Advanced Kit, (Qiagen, Hilden, Germany) according to the manufacturer’s protocol. cel-miR-39 from *Caenorhabditis elegans* (1 nM) was spiked in. In the final step, total RNA (> 18 nucleotides) was eluted using 20 µl of RNase-free water.

##### Reverse transcription and quantitative real-time-PCR

For reverse transcription, the miRCURY LNA RT Kit (Qiagen, Hilden, Germany) was used and quantitative real-time PCR was performed using miRCURY LNA miRNA PCR assays (Appendix A) in a 10 µl reaction containing 3 µL of cDNA (1:30) and a CFX real-time PCR detection system (BioRad, Munich, Germany). Assays were performed in duplicate. The amount of the respective miRNA was normalized to miR-425-3p (previously determined as being stably expressed pre and post training intervention) and cel-miR-39 (spike-in control). A Ct cut-off of 35 was set as the lower limit of detection and the fold change was calculated as (2 − ΔΔ*C*t), which represents the average normalized miRNA expression (2 − ΔΔ*C*t) of the samples in the test group divided by the average normalized miRNA expression (2 − ΔΔ*C*t) of the samples in the control group.

### Data analyses and statistics

After the range-data-plausibility control, outcomes were analysed using pre and post exercise values and absolute pre- to post-differences. Variables continuously monitored during the exercise bouts were processed in their absolute values. All analyses were performed using parametric or non-parametric testing based on the underlying assumptions (data structure, distribution of the variances and variance homogeneity). Between-group differences and pre- to post-changes were determined using omnibus and, in case of a significant omnibus main effect, follow-up post hoc testing. For the statistical calculations, SPSS 23 (SPSS Inc., Chicago, IL, USA) was used. For all statistical analyses, an alpha-error level of 5% was considered a relevant cut-off value for significance testing, *p* values below indicate significant effects.

## Results

### Sample

**Fig. 1:**
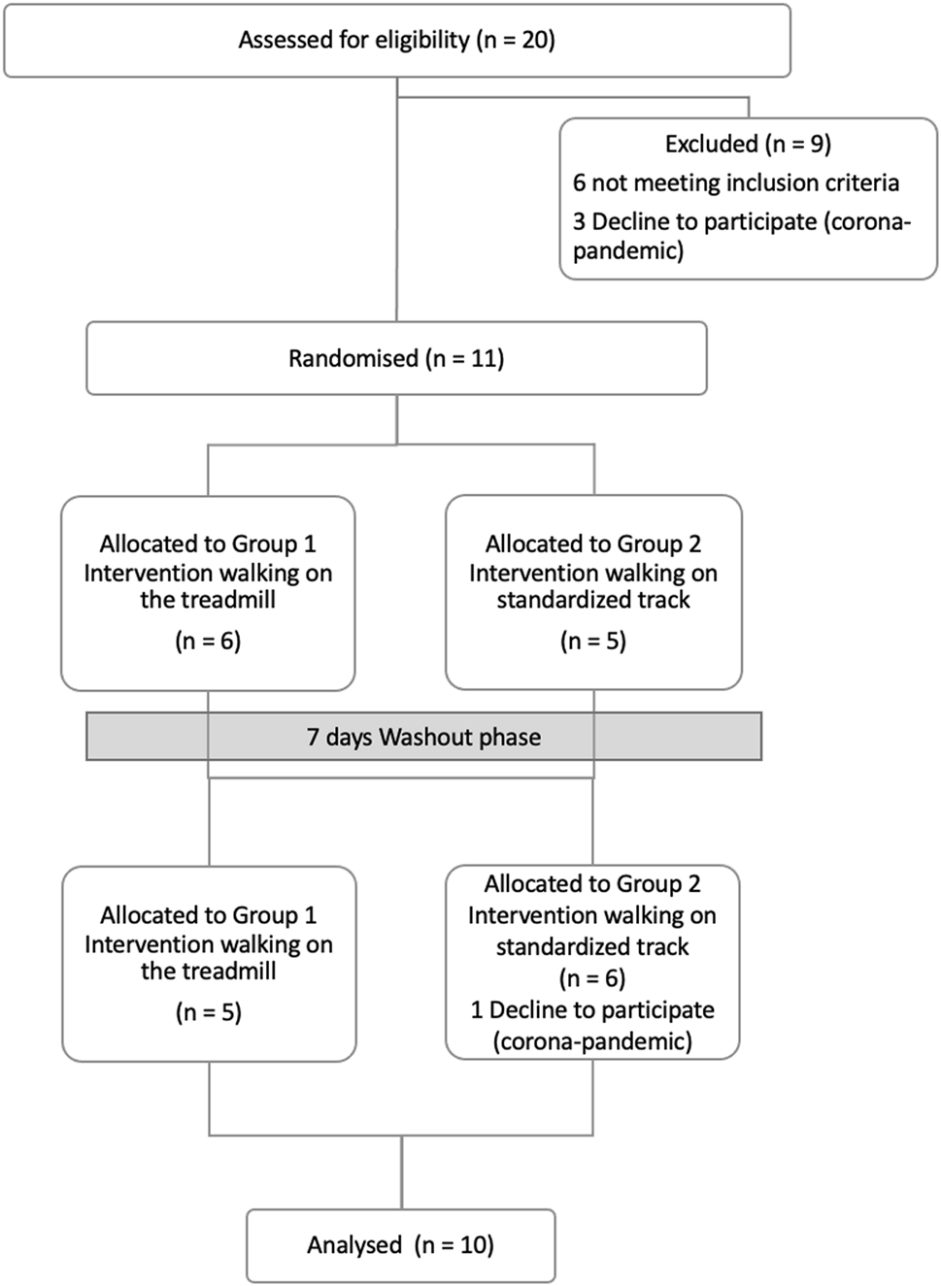
Participant CONSORT flow diagram

The sample consisted of *n* = 10 (*m* = 9, *w* = 1) participants (Fig. 1). The participants were between 63 and 81 years old (mean 72; standard deviation 6.5 years), 164 to 184 cm tall (176 ± 7 cm) and weighed between 49 and 121 kg (83 ± 18 kg). The pain free walking distance was between 150 and 580 m (353 ± 177 m).

The amount of physical activity time averaged 12.5 h per week (standard deviation 7.22 h). The date of diagnosis varied among the patients from January 2012 to December 2018, with secondary diagnosis including coronary artery disease (2 participants), arterial hypertension (6 participants) and celiac disease (1 participant). No participant was smoker during study participation. The ankle–brachial index ranged from 0.6 to 0.85. Based on the ankle–brachial index, 5 patients had a low-grade of PAD and 5 patients a moderate-grade PAD.

### Training outcomes

#### Walking distance and total training time

During the moderate-intensity walking training, a higher total walking distance was covered compared to the vigorous intensive training on the treadmill (*p* = 0.001). On average, the participants walked in the moderate intensity at a self-selected speed of 1.04 m/s on flat ground. The total training time in the LI group was 20 min for all test persons. Training time of the HI training was 17 min (standard deviation 5 min).

#### Lactate concentration

Lactate concentration was increased after vigorous-intensity training (*p* = 0.005). In contrast the lactate concentration after moderate-intensity training was not significantly increased compared to baseline (*p* = 0.179). Pre–post-differences in lactate concentration of both intervention groups were significantly different. The treadmill vigorous-intensity training results in a higher lactate accumulation than the moderate-intensity walking training (1.7 vs. 3.3 mmol/l; *p* = 0.010).

**Fig. 2:**
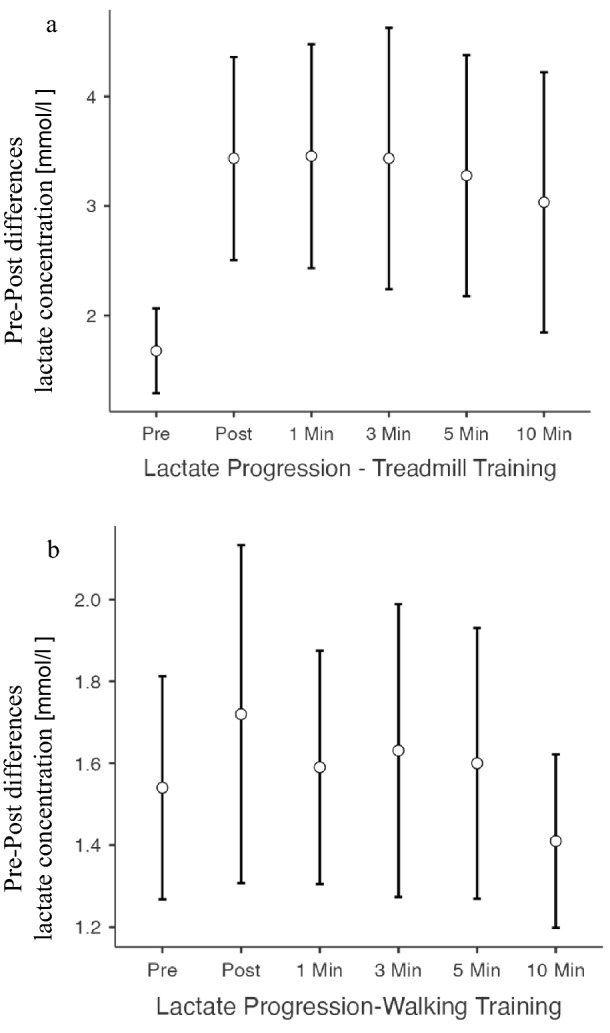
Differences in lactate progression pre-and post-exercise until 10 min post-intervention. Data are displayed as mean and 95% confidence interval. **a** Vigorous-intensity intervention on the treadmill, **b** moderate-intensity intervention with individual selected speed on flat ground

The interventions resulted in two different lactate progressions in the post-exercise phase (Fig. 2). After training with vigorous intensity on the treadmill, none of the test persons reached the baseline lactate concentration 10 min post-exercise. After moderate intensity, walking 8 test persons were able to reach the baseline lactate concentration ± 0.1 mmol/l after 5 min rest and another 2 participants after 10 min.

#### Heart rate

The heart rate was increased during both training interventions (*p* < 0.001). A significant difference between the maximal heart rate during the training interventions occurred. The vigorous-intensity treadmill training resulted in a higher maximal heart rate than the moderate-intensity walking training (125 ± 21 vs. 88 ± 9 bpm; *p* < 0.001). The participants averagely reached 56% of their individual maximal heart rate during the walking training (ranging from 48 to 64%). During the vigorous-intensity training, the participants had a significantly higher percentage heart rate with a mean value of 78% (range from 63 to 95%) of maximal heart rate (*p* < 0.001).

#### miRNAs

**Fig. 3:**
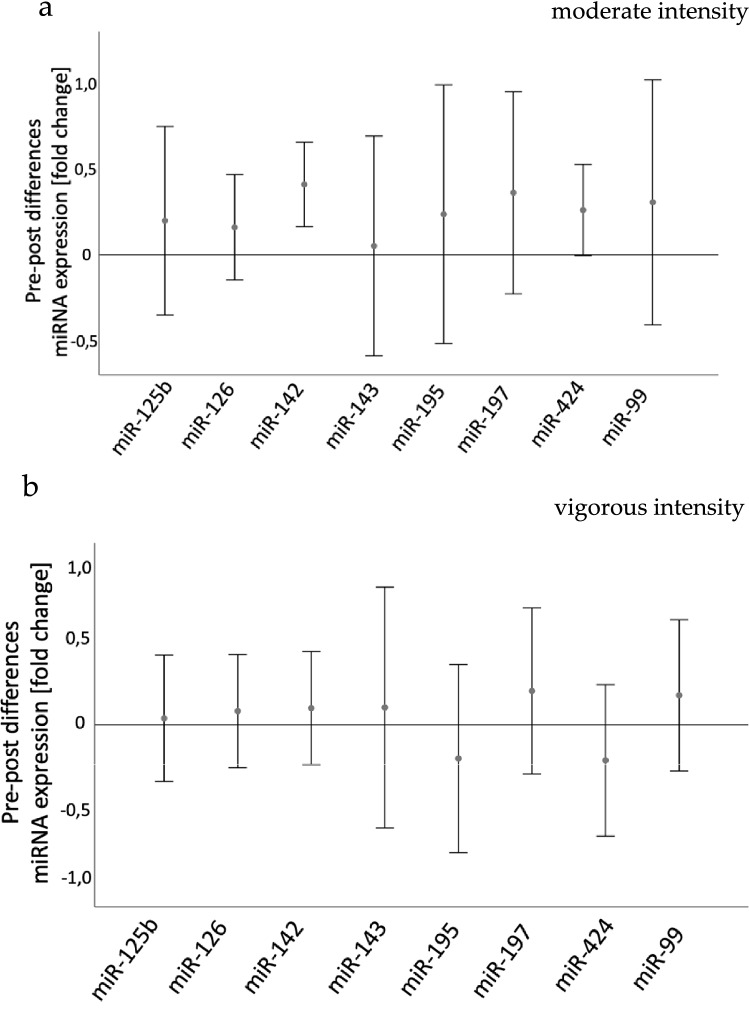
Differences in miRNA expression pre- and post-training. Data are displayed as mean and 95% confidence intervals. **a** Vigorous-intensity training. **b** Moderate-intensity training

Concentrations of eight different training-associated ci-miRNAs were analyzed in each patient before and after moderate or vigorous-intensity intervention. During the moderate-intensity intervention, two of the eight miRNAs were up-regulated: miRNA142-5p (*p* = 0.002) and miRNA-424-5p (*p* = 0.031) (Fig. 3a). None of the miRNA expressions presented significant changes after the vigorous-intensity intervention (Fig. 3b). Omnibus testing indicated no effect of exercise condition on adaptations in miRNA expression (pre–post-difference) (*p* > 0.005). Furthermore, the miRNA-expressions show different pre–post-effect sizes between the observed groups (Cohen’s d: miR-125b-5p d = -0.055; miR-126-5p *d* = – 0.079; miR-142-5p *d* = – 0.696; miR-143-3p *d* = 0.224; miR-195-5p *d* = – 0.306; miR-197-3p *d* = – 0.198; miR-424-5p *d* = – 0.780; miR-99 *d* = – 0.237).

## Discussion

We investigated the concentration of eight circulating miRNAs in patients with peripheral arterial disease in the lower extremities before and after a vigorous- and a moderate-intensity bout of walking exercise. In the vigorous-intensity intervention, a higher anaerobic metabolic response but no significant intervention effect on miRNA expression occurred. Although lactate response was lower, the moderate-intensity intervention led to an upregulation of two miRNA concentrations. Exercise intensity and consecutive metabolic response, indicated by lactate, showed no significant association to miRNA expression. Hypothesis 1, that vigorous-intensity training leads to a higher metabolic response than moderate-intensity training, is thus only partially verified. Hypothesis 2, that moderate-intensity training leads to a different profile of circulating miRNAs compared to high intensity training, is verified.

The moderate-intensity walking training led to lower lactate and maximal heart rate values compared to the vigorous-intensity walking training. The vigorous-intensity intervention was terminated after reaching subjective exertion one time and the distances covered on the treadmill ranged from 671 to 1213 m and thus was shorter than the distance covered in the moderate-intensity walking intervention. The variance in the covered distance until exhaustion underlines the different manifestations of peripheral arterial disease-related impairments, that may result in different metabolic responses during exercise. When comparing the heart rate during interventions to the calculated maximal heart rate, the participants averagely reached 56% of their individual maximal heart rate after the moderate walking training, with a spread of the difference from 48 to 64%. In contrast, after the vigorous-intensity training, the participants had a significantly higher percentage heart rate with a mean value of 78% of HRmax (min: 63%, max: 95%). According to the relative intensity table (Bjarnason-Wehrens et al. 2007), the percentage HR after the moderate-intensity training corresponds to a moderate intensity, whereas the percentage HR after the vigorous-intensity intervention corresponds to an effort described as vigorous. This suggests that the vigorous-intensity intervention represented an intense metabolic stimulus for all test persons despite different walking distances. Likewise, increased lactate concentrations indicate that exhaustive treadmill exercise was a feasible metabolic stimulus whereas a standard walking intervention did not induce a comparable effect. Our study, however, indicates that miRNA expression is not linked to lactate accumulation or exercise intensity (moderate versus vigorous intensity). Since walking distance and duration differed between our interventions as well, further studies are necessary to analyse if these exercise prerequisites impact the effect on circulating miRNA profiles.

In conservative therapy, the effect of exercise on arteriogenesis is considered as an important mechanism. Physical training is thought to have the potential to stimulate neovascularisation in hypoxic and ischaemic tissues, such as the myocardium or peripheral limbs (Guerreiro et al. 2016; Menêses et al. 2017). Arteriogenesis is defined as the growth of functional collateral arteries from pre-existing arterio-arteriolar anastomose (Schaper 2003). Recent studies show that exercise can induce arteriogenesis in humans (Dopheide et al. 2017; Sayed et al. 2010; Nash et al. 1996). To better understand the mechanisms behind this, some studies have linked miRNA expression to arteriogensis (Vogel et al. 2020; Troidl et al. 2009).

The regulation of miRNAs appears to be complex and is certainly not fully understood (Gulyaeva and Kushlinskiy 2016). One possible mechanism could be that the selective uptake of miRNAs by skeletal muscle is stimulated during exercise. Several studies have demonstrated the ability of target cells, such as skeletal muscle, to consume circulating miRNAs (Vickers et al. 2011; Mittelbrunn et al. 2011). Similarly, it has been hypothesised that exercise can cause the release of certain miRNAs from skeletal muscle, e.g. due to muscle damage or through selective release in response to acute or regular exercise (Aoi et al. 2013; Denham and Prestes 2016; Vogel et al. 2019). Resulting changes in circulating miRNA could impact vascular adaptations like vessel growth (Troidl et al. 2020), regulation of perfusion recovery in response to arterial occlusion (Heuslein et al. 2018), neovascularization (Kwast et al. 2019; Pankratz et al. 2015) and vascular smooth muscle proliferation (Hutcheson et al. 2014).

The individual miRNAs are associated with different metabolic reactions, different parts of the human organism and different signalling pathways. MiRNA-142-5p is, among others, associated, with the pathways of VEGF and mTOR signalling, especially in cardiac muscles. This miRNA could be a factor for improvements of the vascular system and arteriogenesis. Moreover, the upregulation of miR‑142‑5p expression is related with the apoptosis in human macrophages by targeting TGF‑β2, this effect could play an important role in the progression of atherosclerosis (Xu et al. 2015). Another study associated miRNA 142-5p with endurance training in generally in healthy adults (Sieland et al. 2021).

MiRNA-424-5p is associated with the expression of selected endothelial angiogenetic mediators in response to these growth factors (VEGF and bFGF) and is, therefore, considered as a “hypoximiR” (Chamorro-Jorganes et al. 2011). This concludes that miR-424-5p could play an important role in regulating cell-intrinsic angiogenic activities of endothelial cells. In another study endurance training with blood flow restriction stimulate the expression of miRNA-424-5p. This result is supported by studies reporting that BFR resistance training improved vascular endothelial function and peripheral blood circulation with an increased expression of lactate, norepinephrine, vascular endothelial growth factor and growth hormones (Shimizu et al. 2016). Reduced blood flow external or due to disease can cause changes in miRNA-424-5p expression. Our study extends these findings on healthy subjects in so far that lower intensity exercise seems feasible to induce comparable miRNA adaptations in patients. One explanation might be the reduced blood flow due to peripheral stenosis.

A study limitation is the small number of subjects as a result to the corona pandemic. Individuals with PAD tend to have many comorbidities that may be related to the observed outcomes. Some comorbidities affect similar cardiovascular and metabolic mechanisms as PAD. Examples include diabetes mellitus, coronary artery disease, heart failure, arterial hypertension and celiac disease.

Our results are consistent with the proposed epigenetic potential of activity interventions that may alter miRNA expression. Since each miRNA is predicted to have many targets, we did not identify any direct association with other measures. Therefore, we postulate that further studies are necessary to adjust the intensity and extent of training to gain more information about the relationship between individual miRNAs, Peripheral arterial disease and human metabolism. This could be an added value (1) in training control and (2) in diagnostic screening of miRNAs to stratify responders and non-responders for individualization of treatment in PAD patients.

## Conclusion

In conclusion, walking training induces changes in circulating miRNA expression in patients with peripheral arterial disease. These changes only occur during moderate walking training for at least 20 min. Future studies are needed to find out whether (1) longer intensive training sessions, e.g. as interval training, and (2) several training sessions over a longer training period induce changes in miRNAs-expression and whether (3) these changes in miRNAs play a physiological role by which walking training positively influences whole-body metabolism in patients with peripheral arterial occlusive disease and favours disease progression of recovery.

## Supplementary Information

Below is the link to the electronic supplementary material.

Supplementary file1 (DOCX 13 KB)

## Abbreviations

bFGF: Basic fibroblast growth factor
BFR: Blood flow restriction
BPM: Beats per minute
ci-miRNA: Circulating microRNA
EDTA: Ethylenediaminetetraacetic acid
HR: Heart rate
LC: Lactate concentration
miRNA: MicroRNA
mTOR: Mechanistic target of rapamycin
OD: Optical density
PAD: Peripheral artery disease
PCR: Polymerase chain reaction
RNA: Ribonucleic acid
SD: Standard deviation
UTR: Untranslated region
VGEF: Vascular endothelial growth factor
