# Supplementary material for: Changes in miRNA expression in patients with peripheral arterial vascular disease during moderate- and vigorous-intensity physical activity
Source: Eur J Appl Physiol. 2022 Nov 23;123(3):645–54. doi: 10.1007/s00421-022-05091-2 (PMC9684818; doi:10.1007/s00421-022-05091-2)
Supplement: Supplementary file 1 — Supplementary file1 (DOCX 13 KB) [file 421_2022_5091_MOESM1_ESM.docx]

Appendix A

A **Table A1.** miRCURY LNA miRNA PCR assays for qRT-PCR.

| miRNA | Assay no. | sequence |
| --- | --- | --- |
| hsa-miR-197-3p | YP00204380 | 5′UUCACCACCUUCUCCACCCAGC |
| hsa-miR-143-3p | YP00205992 | 5′UGAGAUGAAGCACUGUAGCUC |
| hsa-miR-424-5p | YP00204736 | 5′CAGCAGCAAUUCAUGUUUUGAA |
| hsa-miR-142-5p | YP00204722 | 5′CAUAAAGUAGAAAGCACUACU |
| hsa-miR-126-5p | YP00206010 | 5′CAUUAUUACUUUUGGUACGCG |
| hsa-miR-199a-5p | YP00204494 | 5′CCCAGUGUUCAGACUACCUGUUC |
| hsa-miR-125b-5p | YP00205713 | 5′UCCCUGAGACCCUAACUUGUGA |
| hsa-miR-195-5p | YP00205869 | 5′UAGCAGCACAGAAAUAUUGGC |
